# The development of an evidence-based clinical checklist for the diagnosis of anterior knee pain

**DOI:** 10.4102/sajp.v73i1.353

**Published:** 2017-03-31

**Authors:** Dominique C. Leibbrandt, Quinette Louw

**Affiliations:** 1 Department of Physiotherapy/FNB-3D Movement Analysis Laboratory, Faculty of Medicine and Health Sciences, University of Stellenbosch, South Africa

## Abstract

**Background:**

Anterior knee pain (AKP) or patellofemoral pain syndrome is common and may limit an individual’s ability to perform common activities of daily living such as stair climbing and prolonged sitting. The diagnosis is difficult as there are multiple definitions for this disorder and there are no accepted criteria for diagnosis. It is therefore most commonly a diagnosis that is made once other pathologies have been excluded.

**Objectives:**

The aim of this study was to create an evidence-based checklist for researchers and clinicians to use for the diagnosis of AKP.

**Methods:**

A systematic review was conducted in July 2016, and an evidence-based checklist was created based on the subjective and objective findings most commonly used to diagnose AKP. For the subjective factors, two or more of the systematic reviews needed to identify the factor as being important in the diagnosis of AKP.

**Results:**

Two systematic reviews, consisting of nine different diagnostic studies, were identified by our search methods. Diagnosis of AKP is based on the area of pain, age, duration of symptoms, common aggravating factors, manual palpation and exclusion of other pathologies. Of the functional tests, squatting demonstrated the highest sensitivity. Other useful tests include pain during stair climbing and prolonged sitting. The cluster of two out of three positive tests for squatting, isometric quadriceps contraction and palpation of the patella borders and the patella tilt test were also recommended as useful tests to include in the clinical assessment.

**Conclusion:**

A diagnostic checklist is useful as it provides a structured method for diagnosing AKP in a clinical setting. Research is needed to establish the causes of AKP as it is difficult to diagnose a condition with unknown aetiology.

## Introduction

Knee pain affects about 70% of clients visiting the community health centres in the Western Cape (Parker & Jelsma 2010). This alarming occurrence of knee problems is associated with moderate to high levels of disability.

Anterior knee pain (AKP) or patellofemoral pain syndrome (PFPS) frequently affects the knee joint and impairs functional ability (Parker & Jelsma 2010).

The international incidence has been reported to be 25%–43% in sports injury clinics (Callaghan & Selfe 2007; Witvrouw et al. 2000). AKP has a tendency to become chronic, and it has been estimated that 91% of patients diagnosed with AKP still experience symptoms four years after its onset. AKP is particularly common in adolescents, between the ages of 12 and 17 years (Rathleff et al. 2013), and may limit an individual’s ability to perform common activities of daily living such as stair climbing and prolonged sitting (Nunes et al. 2013).

AKP is thought to be multifactorial in origin (Aminaka & Gribble 2008). It also has the tendency to become chronic, especially in active individuals, adding an additional aspect of complexity to the treatment (Collins et al. 2012). There is agreement among recent reviews that conservative approaches are the preferred choice of treatment for AKP (Collins et al. 2012; McCarthy & Strickland 2013). Surgical options such as distal realignment of the extensor mechanism, lateral retinacular release or debridement are generally only considered when conservative methods have failed or in the case of severe instability (McCarthy & Strickland 2013).

The aetiology of AKP is not well understood. In addition, the aetiology may differ depending on whether symptoms are acute or chronic. There are a variety of pathways that could result in ongoing pain (psychological, pathophysiological, mechanical). However, the onset of the condition is hypothesised to involve excessive joint stress during activities that load the flexed knee joint. This patellofemoral joint stress is then transmitted through the cartilage, thereby exciting nociceptors in subchondral bone resulting in pain (Fulkerson 2002). Over time, this joint stress may result in articular cartilage pathology (Powers et al. 2014).

There are many definitions and synonyms for AKP. It is often used as an umbrella term for pathologies that cannot be classified as anything else, and therefore can include a variety of different pathologies. The term has been used interchangeably with PFPS, chondromalacia patellae, runner’s knee, patellofemoral joint dysfunction and patella arthralgia (Collins et al. 2012; Cook et al. 2010; Lake & Wofford 2011; Nunes et al. 2013). For the purpose of this article, we will be using the term ‘anterior knee pain’.

Appendix 1 illustrates the range of definitions reported in systematic reviews.

The multiple definitions of AKP make accurate and standardised clinical diagnosis a challenging task for clinicians.

AKP is frequently defined as retropatellar or peripatellar pain, of more than three months duration, in the absence of intra-articular pathology, that is aggravated by activities that load a flexed knee joint (Crossley et al. 2001; Harvie, O’Leary & Kumar 2011; Nunes et al. 2013; Prins & van der Wurff 2009). The diagnosis of AKP is most commonly made based on the definition as well as the exclusion of other pathologies. However, this diagnostic procedure is vague and difficult to reproduce in a clinical setting.

The aim of this study was to create an evidence-based checklist for researchers and clinicians to use for the diagnosis of AKP.

## Methods

### Study selection criteria

English-only studies reporting on the clinical diagnostic tests for AKP were considered for inclusion. Due to the abundance of literature on AKP, only systematic reviews were eligible for inclusion.

Studies describing the subjective information used for the diagnosis of AKP, such as the age of the patient, the duration of the symptoms, aggravating activities and previous history of trauma or other known knee injuries, were considered for inclusion.

Studies describing objective clinical tests used for the diagnosis of AKP were included. Radiographic procedures such as MRIs were excluded as these procedures cannot form part of a physiotherapy clinical assessment. For the same reason, arthroscopic procedures were also excluded.

The subjects of the studies included both genders. Exclusions were for studies that may have incorporated diagnoses of Osgood-Schlatter and osteoarthritis in participants younger than 18 years or older than 40 years. In addition, studies portraying knee abnormalities such as patella subluxation or intra-articular pathology were also omitted.

#### Search strategy

Publications from inception to July 2016, located in PubMed, Ebscohost (MEDLINE, CINAHL, SportDiscuss), Scopus and Science Direct, were accessed in library databases at the Medical Library at Stellenbosch University during July 2016.

The keywords used by the researcher (D.L.) in all the searches were: ‘anterior knee pain’, ‘patellofemoral pain syndrome’, ‘diagnosis’, ‘clinical tests’ and ‘systematic reviews’. Searches were database-specific with MeSH terms for ‘patellofemoral pain syndrome’ used in search engines such as PubMed.

PRISMA Guidelines were followed with the reviewer (D.L.) screening the titles and abstracts of the first hits and consulting with the second reviewer (Q.L.) as needed. Both reviewers retrieved all potential complete texts independently and used the same criteria to decide which ones were relevant for inclusion in the review after having considered possible discrepancies in the texts. The individual diagnostic studies within the included reviews were then analysed.

### Methodological quality appraisal

A clinical appraisal tool (CAT) for systematic reviews was used for the appraisal of included studies. This CAT comprises 10 questions assessing the methodological quality of the study and validity of the findings.

This CAT, as well as a detailed explanation of the criteria, can be found on the BMJ website (http://clinicalevidence.bmj.com/x/set/static/ebm/toolbox/665052.html) and is present in Appendix 2.

### Development of a diagnostic checklist

An evidence-based checklist was created based on the subjective and objective findings. For the subjective factors, two or more of the systematic reviews were needed to identify the factor as being important in the diagnosis of AKP. For the objective factors, two or more of the reviews were needed to recommend the test based on either a sensitivity (more than 70%) or a positive likelihood ratio (more than 5). A positive likelihood ratio of between 0 and 5 is considered to generate small but clinically important changes in probability (Nijs, Van Geel & Van de Velde 2006). Clusters of tests found to improve diagnosis in any of the included reviews were also considered for the checklist.

## Results

**FIGURE 1:**
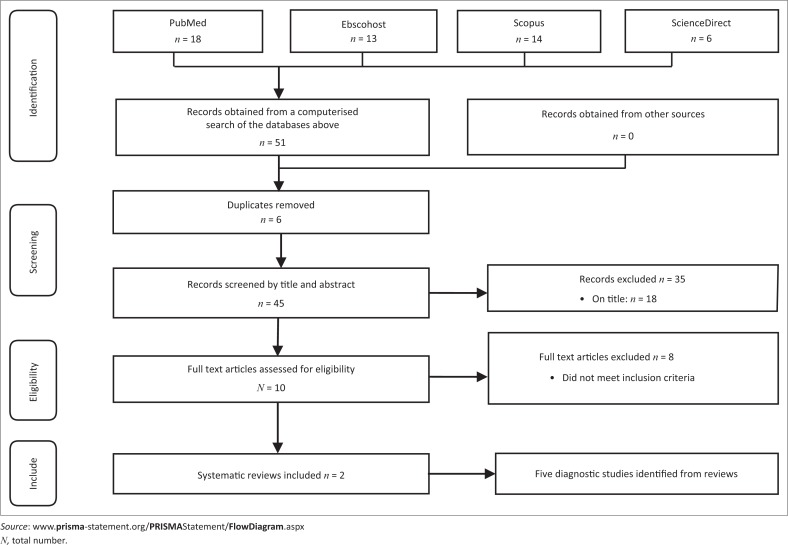
PRISMA flow diagram of literature search.

Two systematic reviews (Cook et al. 2012; Nunes et al. 2013), consisting of nine different diagnostic studies, were identified by our search methods. Of the nine diagnostic studies, four full texts were excluded as they used arthroscopic surgery for diagnosis and not clinical tests. A PRISMA flow chart is given in Figure 1.

**FIGURE 2:**
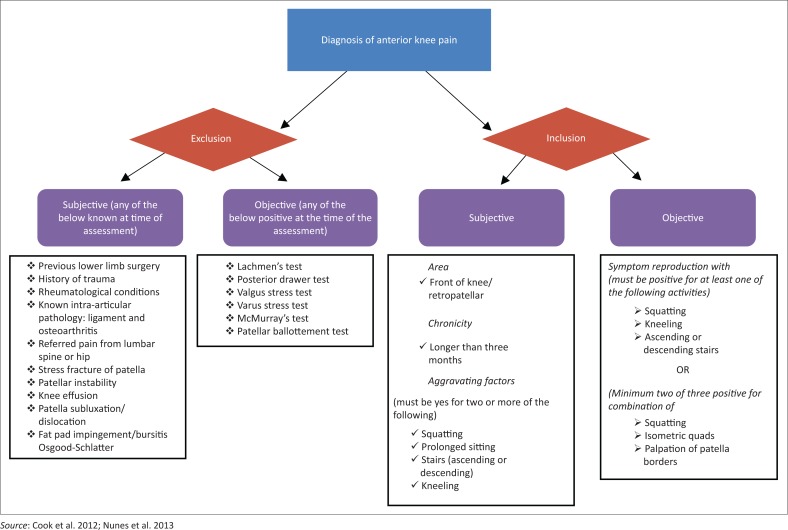
Flowchart demonstrating the process of diagnosis for anterior knee pain.

The final checklist is presented in Appendix 3. Based on these studies, initial information that should be included in the subjective assessment includes age, area of pain, duration of symptoms, previous history of lower limb trauma or surgery and common aggravating factors. A flow chart of the diagnostic procedure is given in Figure 2.

As AKP is still largely a diagnosis of exclusion, patients should not be diagnosed with AKP if they are known to have any of the following pathologies: osteoarthritis, rheumatoid arthritis, patella fractures, patella subluxation and dislocation, fat pad impingement or bursitis, growth disorders such as Osgood-Schlatter, intra-articular pathology, patellar tendinitis, or referred pain from the lumbar spine or hip (Cook et al. 2010; Haim et al. 2006; Nijs et al. 2006; Sweitzer et al. 2010).

Objective tests can be divided into functional clinical tests, manual tests and exclusion of intra-articular pathologies.

Table 1 summarises the accuracy of commonly used diagnostic tests for AKP. Clinical functional tests that most commonly reproduce symptoms in patients with AKP are squatting, kneeling, stair climbing and prolonged sitting. Squatting is the most accurate functional test with a sensitivity of 91%. Kneeling, stair ascent or descent and prolonged sitting follow with sensitivities of 84%, 75% and 72%, respectively (Cook et al. 2010; Haim et al. 2006; Näslund et al. 2006; Nijs et al. 2006; Sweitzer et al. 2010).

It has been suggested that patients should present with pain in two or more of these activities in order to be diagnosed with AKP (Cook et al. 2012).

Of the manual tests considered, only the patella compression test (sensitivity of 83%) and the patella tilt test (likelihood ratio = 5.4) can be recommended as diagnostic tests for AKP (Haim et al. 2006; Näslund et al. 2006; Sweitzer et al. 2010).

On clinical appraisal of the two included systematic reviews (Cook et al. 2012; Nunes et al. 2013), both studies achieved scores of 8/10, or 80%. Therefore, these reviews can be considered to be of high methodological quality. Table 2 shows the scoring according to the CAT.

## Discussion

In this article, we created a standardised method for the diagnosis of AKP based on a systematic review of the evidence. Diagnosis of AKP is based on the area of pain, age, duration of symptoms, common aggravating factors, manual palpation, and exclusion of other pathologies.

**TABLE 1:** Accuracy of diagnostic tests.

Test	Sensitivity	Specificity	LR+	LR˗	PV+	PV˗
Squatting	91	50	1.8	0.2	79	74
Kneeling	84	50	1.7	0.3	79	61
Stairs – ascending and descending	75	43	1.3	0.6	73	46
Prolonged sitting	72	57	1.7	0.5	77	50
Patella tilt test	43	92	5.4	0.6	93	40
Patella compression test	83	18	1.0	1.0	63	38

*Source*: Cook et al. 2010; Haim et al. 2006; Näslund et al. 2006; Nijs et al. 2006; Sweitzer et al. 2010

LR, likelihood ratio; PV, predictive value.

**TABLE 2:** Quality of evidence.

Study	Cook et al. 2011			Nunes et al. 2013		
	
SR quality criteria	Yes	No	Can’t tell	Yes	No	Can’t tell
1	×	-	-	×	-	-
2	-	×	-	-	×	-
3	×	-	-	×	-	-
4	×	-	-	×	-	-
5	×	-	-	×	-	-
6	×	-	-	×	-	-
7	×	-	-	×	-	-
8	×	-	-	×	-	-
9	×	-	-	×	-	-
10	-	×	-	-	×	-

**Total**	**8/10**	**-**	**-**	**8/10**	**-**	**-**

*Source*: http://clinicalevidence.bmj.com/x/set/static/ebm/toolbox/665052.html

SR, systematic review.

**TABLE 3:** Most accurate tests for exclusion of intra-articular pathology.

Test	Structure	Sensitivity (%)	Specificity (%)
Lachmen’s	ACL	85	94
Anterior drawer	ACL	92	91
Posterior drawer	PCL	51–100	99
Valgus stress	MCL	86–96	Not reported
Varus stress	LCL	25	Not reported
Pivot shift	Meniscus	24	98
McMurray’s	Meniscus	16–58	77–98
Apley’s grind	Meniscus	13–16	80–90
Patella ballottement	Effusion	32	100

*Source*: Benjaminse et al. 2006; Day et al. 2009; Malanga et al. 2003; Nijs et al. 2006

AKP can be defined as pain in the infrapatellar or retropatellar regions, in the absence of intra-articular pathology, that restricts activities of daily living that require knee flexion such as ascending or descending stairs, squatting and prolonged sitting (Cook et al. 2010; Haim et al. 2006; Näslund et al. 2006; Nijs et al. 2006; Sweitzer et al. 2010).

The subjective examination is important in the diagnosis of AKP. The interview should localise the pain, define the timing of onset and determine acute versus chronic versus overuse (Pećina & Bojanić 1993). This information is important as it helps the clinician to rule out competing diagnoses. Extensor mechanism dysfunction is most commonly as a result of chronic repetitive trauma. AKP can also be patella subluxation or dislocation, ruptured patella or quad tendons. AKP during rest is often indicative of chondral lesions or dysfunctions (Post 1999; Smith et al. 2010).

A systematic review by Nunes et al. (2013) looked at five studies, that in total analysed 25 tests commonly used to diagnose AKP. The review concluded that there is no consistent evidence regarding the accuracy of commonly used diagnostic tests for AKP. However, the patellar tilt test (Haim et al. 2006) and the pain during squatting test (Cook et al. 2010) showed a strong tendency towards the PFPS diagnosis. The pain during squatting test demonstrated the highest sensitivity.

The other systematic review that was acquired through our search procedures (Cook et al. 2011) included nine studies; however, four were excluded as they made use of arthroscopy. The review included a variety of tests used to reproduce AKP including functional tests, patella mobility tests, special tests and the Q angle test. Of these the functional tests, in particular squatting, stair climbing and prolonged sitting, demonstrated the highest accuracy.

Five diagnostic studies were identified from these two reviews (Cook et al. 2010; Haim et al. 2006; Näslund et al. 2006; Nijs et al. 2006; Sweitzer et al. 2010).

Nijs et al. (2006) investigated the validity of five clinical tests for AKP, including the vastus medialis coordination test, the patellar apprehension test, Waldron’s test, Clarke’s test and the eccentric step test.

In this study, the vastus medialis and patellar apprehension tests had a ratio of 2.26 and the eccentric step test scored 2.34. Waldron’s test and Clarkes’s test both scored below 2, thus questioning their validity. Limitations of the study included inability to standardise the amount of force used, the tests were performed in isolation and in reality these tests would be combined with other tests as part of a full subjective and objective clinical evaluation. The order of the tests also should have been standardised. Based on our criteria for inclusion, none of these tests is accurate enough to be considered for diagnosis.

Cook et al. (2010) explored the diagnostic accuracy of physical tests and functional activities commonly used to diagnose AKP. Clusters of functional findings and physical examination tests were also tabulated to determine combinations that improved diagnostic accuracy. Patients with intra-articular pathology were excluded. Measures used were manual compression of kneecap against femur (1) during rest and (2) during an isometric knee contraction, palpation of the posterio-medial and posterio-lateral borders of the patella, resisted isometric quadriceps femoris muscle contraction, squatting, stair climbing, kneeling and prolonged sitting. These measure were investigated as they are routinely used to measure AKP even though very few of these measures have been investigated for accuracy. The authors found that clusters may marginally improve accuracy. The cluster of two out of three positive tests for squatting, isometric quadriceps contraction and palpation of the patella borders scored the highest with a positive likelihood ratio of 4. The authors recommended the use of this cluster of tests to diagnose AKP in a clinical assessment. Individually, squatting, palpation, stepping down and the patella tilt test were recommended as useful tests to include in the clinical assessment.

Sweitzer et al. (2010) investigated the accuracy of patella mobility tests including superior-inferior patellar mobility, medial-lateral patellar mobility, patellar tendon mobility and patellar inferior pole tilt. However, all of these tests demonstrated poor sensitivity (19%–63%) as well as positive likelihood ratios (1.4–1.9) and have therefore not been included in our checklist.

In a study by Näslund et al. in 2006, a physiotherapist and an orthopaedic surgeon examined 80 patients clinically diagnosed with AKP and referred for physiotherapy. The examination included a case history and a clinical examination. The four tests used in the clinical examination were the patella compression test, medial and lateral tenderness on extension, passive gliding of the patella and the Q angle test. The results indicated that the compression test demonstrated the highest sensitivity (83%), but none of the tests could predict findings seen in radiographic examinations. The authors suggested that the Q angle test can no longer be considered a reliable test in diagnosing AKP, as it shows great inter- and intra-observer variability. This is in agreement with a recent systematic review of prospective studies that demonstrated that the Q angle is not a risk factor for AKP, thus questioning its relevance (Smith, Hunt & Donell 2008). The authors (Näslund et al. 2006) suggested the AKP is still ultimately a diagnosis of exclusion as it is a term used for knee pain that can be attributed to multiple causes. Therefore, more research on pathophysiology needs to be done.

A very important aspect of diagnosis for AKP is the exclusion of intra-articular pathologies. These include ligaments such as ACL, PCL, MCL and LCL and the meniscii (medial and lateral). The most accurate tests to achieve this have been given in Table 3 (Benjaminse, Gokeler & van der Schans 2006; Day, Fox & Paul-Taylor 2009; Malanga et al. 2003; Nijs et al. 2006). Based on this, we have chosen to include the anterior drawer test, the posterior drawer test, the valgus stress test, the varus stress test, McMurray’s test and the patellar ballottement test in our checklist for the purpose of exclusion.

The two reviews used for the creation of our evidence-based checklist were both of high quality. The reviews evaluated the quality of the included studies and took this into consideration when making the recommendations. Consequently, we can be confident that the checklist is based on high-quality evidence.

Nevertheless, in order to improve on this evidence, it is necessary to establish possible causes of AKP. Causes are believed to be multifactorial, and diagnosis is still largely a diagnosis of exclusion in a specific population of younger active people. Subgroups of individuals with AKP and aetiology may vary.

## Conclusion

AKP can be defined as retro- or peri-patellar pain, of more than three months duration, in the absence of intra-articular pathology, that is aggravated by activities that load a flexed knee joint (Crossley et al. 2001; Harvie et al. 2011; Nunes et al. 2013; Prins & van der Wurff 2009). The diagnosis of AKP is made based on the definition as well as the exclusion of other pathologies. There are many clinical tests used to diagnose AKP; however, there is no standard method to diagnose AKP and many of the tests are not accurate. A diagnostic checklist is useful as it provides a structured method for diagnosing AKP in a clinical setting. Research is needed to establish the causes of AKP as it is difficult to diagnose a condition with unknown aetiology.

## Appendix 1

**TABLE 1-A1:** Definitions and synonyms for AKP.

Crossley et al. 2001	PFPS AKP	An umbrella term used to encompass all anterior or retropatellar pain in the absence of other specific pathology. All pathologies that may manifest as anterior or retropatellar pain.
Harvie et al. 2011	PFPS	Diffuse retro/peripatellar pain, aggravated with activities which load the patellofemoral joint, such as climbing stairs, squatting, running and prolonged sitting.
Aminaka & Gribble 2005	PFPS	A condition presenting with anterior knee pain or pain behind the patella (retropatella). It is commonly experienced during running, squatting, stair climbing, prolonged sitting and long sitting.
Cook et al. 2011	Chondromalacia patellaePFPSAKP	Old term used for PFPS. Anterior knee pain including the patella, but not including tibiofemoral or peripatellar structures. Anterior knee pain of more than three months duration, aggravated by prolonged sitting, squatting, and ascending and descending stairs. All pain at the front of the knee.
Nunes et al. 2013	PFPS	In the absence of other intra-articular disorders, there is currently consensus that anterior knee pain, which limits activities of daily living that demand knee flexion such as climbing and descending stairs, squatting or remaining seated. Synonyms include chondromalacia patellae, patella arthralgia and patella pain.
Lake & Wofford 2011	Runner’s kneePFPS	Synonym for PFPS as it is common in runners and other endurance athletes. AKP characterised by diffuse anterior knee pain, aggravated with specific activities that heighten the compressive loading forces across the patellofemoral joint including ascending and descending stairs, squatting and prolonged sitting.
Collins et al. 2012	AKP	Synonym for PFPS. Chronic musculoskeletal overuse condition of the knee that affects an individual’s ability to perform routine daily activities such as stair ambulation, walking and running, and thus impacts on work-related activities and participation in physical activity.
Barton, Webster & Menz 2008	PFPS	AKP of insidious onset defined as the presence of pain in the retropatellar or peripatellar region during tasks that increase patellofemoral joint loading, such as walking, running, negotiating stairs, squatting, prolonged sitting and kneeling. Anterior knee pain or retropatellar pain in the absence of other specific pathology.
Heintjes et al. 2003	PFPS	Retropatellar pain (behind the kneecap) or peripatellar pain (around the kneecap) when ascending or descending stairs, squatting or sitting with flexed knees.
Prins & Van der Wurff 2009	PFPS	The remainder of knee pain cases after intra-articular pathologies, patella tendonopathies, peripatellar bursitis, plica syndrome, Sinding-Larsen Johnson and Osgood-Schlatter have been excluded.
Callaghan & Selfe 2012	PFPSAKP	The clinical presentation of knee pain related to changes in the patellofemoral joint. Pain at the front of the knee, separate from arthritis. Gradual onset of knee pain with none of the features associated with other knee injuries or diseases. Pain at the front of the knee, used synonymously with PFPS.
Waryasz & McDermott 2008	PFPSAKP	A variety of pathologies or anatomical abnormalities leading to a certain type of AKP. Broader term for all pathologies causing pain at the front of the knee, including referred pain from the lumbar spine or hip.
Heintjes et al. 2004	PFPSRetropatellar pain	A common complaint in adolescents and young adults, most frequently characterised by diffuse peripatellar and retropatellar localised pain, typically provoked by ascending or descending stairs, squatting and sitting with flexed knees for prolonged periods of time. Retropatellar pain in which no cartilage damage is evident. A self-limiting condition of the knee, which includes cartilage damage.
Lankhorst, Bierma-Zeinstra & Van Middelkoop 2012	PFPSAKP	A condition of anterior knee pain. Pain in or around the patella. This pain increases after prolonged sitting, squatting, kneeling and stair climbing. Covers all problems related to the anterior part of the knee.

AKP, anterior knee pain; PFPS, patellofemoral pain syndrome.

## Appendix 2

**TABLE 1-A2:** Framework for assessing systematic reviews.

General systematic review quality criteria	Yes	No	Can’t tell
Does the SR explicitly report and perform a comprehensive and reproducible literature search?			
Does the SR formulate a clearly focused question?			
Does the SR’s methods section explicitly state the basis for inclusion or exclusion of primary RCTs?			
Does the SR report data from primary RCTs (e.g. size, interventions used, results from individual RCTs)			
Does the SR assess the methodological quality of primary studies, and take these into account where necessary?			
Meta-analysis: does the SR combine primary studies appropriately?			
Meta-analysis: does the SR state how results are combined statistically?			
Meta-analysis: does the SR report absolute numbers as well as appropriate summary statistics?			
Does the SR discuss the reasons for any variations or heterogeneity between individual RCTs and overall results?			
Does the SR report on the clinical relevance or importance of the results?			

RCT, randomised controlled trial; SR, systematic review.

## Appendix 3

**Checklist for diagnosis of anterior knee pain.**

**SUBJECTIVE INFORMATION:**

**Table:**

**Age** (must be yes)	YES	NO

**Table:**

14–50^1,2,3,4,5^		

**Area** (must be yes)

**Table:**

Front of knee or retropatella^1,2,3,4,5^		

**Chronicity**

**Table:**

Longer than three months^1,3,5^			
Aggravated by (must be yes for two or more of the following)			
Squatting^1,2,3,4,5^			
Prolonged sitting^1,2,3,4,5^			
Stairs (ascending or descending)^1,2,3,4,5^			
Kneeling^1,2,3,4,5^			

Excluded if any of the below is known

**Table:**

Previous lower limb surgery^1,3,5^			
History of trauma^1,3,5^			
Rheumatological conditions^1,3,5^			
Known intra-articular pathology: ligament and osteoarthritis^1,2,3,4,5^			
Patellar instability^1,4^			
Knee effusion^1,5^			
Patella subluxation/dislocation^1,5^			
Fat pad impingement/bursitis^3,5^			
Osgood–Sclatter^1,3^			

**OBJECTIVE TESTS:**

Symptom reproduction with (must be positive for at least one of the following activities)

**Table:**

Squatting^1,2,3,4,5^			
Kneeling^1,2,3,4,5^			
Ascending or descending stairs^1,2,3,4,5^			
Positive for at least one of the following			
Patella compression test^1,4^			
Patella tilt test^1,4^			

**OR**

(Minimum two out of three) positive for combination of

**Table:**

Squatting^3^			
Isometric quads^3^			
Palpation of patella borders^3^			

**Excluded if positive for**

**Table:**

Lachmen’s test^6,7,8^	ACL		
Posterior drawer test^6,8^	PCL		
Valgus stress test^6,8^	MCL		
Varus stress test^6,8^	LCL		
McMurray’s test^6,8^	MENISCUS		
Patellar ballottement test^5^	Effusion		

## References

[CIT0001] Aminaka N. & Gribble P, 2005, ‘A systematic review of the effects of therapeutic taping on patellofemoral pain syndrome,’ *Journal of Athletic Training* 40(4), 341–351. 16404457 PMC1323297

[CIT0002] Aminaka N. & Gribble P.A, 2008, ‘Patellar taping, patellofemoral pain syndrome, lower extremity kinematics, and dynamic postural control’, *Journal of Athletic Training* 43(1), 21–28. 18335009 10.4085/1062-6050-43.1.21 PMC2231393

[CIT0003] Barton C.J., Webster K.E. & Menz H.B, 2008, ‘Evaluation of the scope and quality of systematic reviews on nonpharmacological conservative treatment for patellofemoral pain syndrome,’ *Journal of Orthopaedic & Sports Physical Therapy* 38(9), 529–541. 10.2519/jospt.2008.2861 18758046

[CIT0004] Benjaminse A., Gokeler A. & Van der Schans C.P, 2006, ‘Clinical diagnosis of an anterior cruciate ligament rupture: A meta-analysis’, *Journal of Orthopaedic & Sports Physical Therapy* 36(5), 267–288. 10.2519/jospt.2006.2011 16715828

[CIT0005] Callaghan M.J. & Selfe J, 2007, ‘Has the incidence or prevalence of patellofemoral pain in the general population in the United Kingdom been properly evaluated?’, *Physical Therapy in Sport* 8(1), 37–43. 10.1002/14651858.cd006717.pub2

[CIT0006] Callaghan M.J. & Selfe J, 2012, ‘Patellar taping for patellofemoral pain syndrome in adults,’ *The Cochrane Library* 4, CD006717. 10.1002/14651858.CD006717.pub2 PMC11831247 22513943

[CIT0007] Collins N.J., Bierma-Zeinstra S.M., Crossley K.M., Van Linschoten R.L., Vicenzino B. & Van Middelkoop M, 2012, ‘Prognostic factors for patellofemoral pain: A multicentre observational analysis’, *British Journal of Sportsmedicine* 47, 227–233. 10.1136/bjsports-2012-091696 23242955

[CIT0008] Cook C., Hegedus E., Hawkins R., Scovell F. & Wyland D, 2010, ‘Diagnostic accuracy and association to disability of clinical test findings associated with patellofemoral pain syndrome’, *Physiotherapy Canada* 62(1), 17–24. 10.3138/physio.62.1.17 21197175 PMC2841549

[CIT0009] Cook C., Mabry L., Reiman M.P. & Hegedus E.J, 2012, ‘Best tests/clinical findings for screening and diagnosis of patellofemoral pain syndrome: A systematic review,’ *Physiotherapy* 98(2):93–100. 22507358 10.1016/j.physio.2011.09.001

[CIT0010] Crossley K., Bennell K., Green S. & McConnell J, 2001, ‘A systematic review of physical interventions for patellofemoral pain syndrome,’ *Clinical Journal of Sport Medicine* 11(2), 103–110. 11403109 10.1097/00042752-200104000-00007

[CIT0011] Day R.J., Fox J.E. & Paul-Taylor G, 2009, *Neuromusculoskeletal clinical tests: A clinician’s guide*, Elsevier Health Sciences Cardiff University, Cardiff.

[CIT0012] Fulkerson J.P, 2002, ‘Diagnosis and treatment of patients with patellofemoral pain’, *The American Journal of Sportsmedicine* 30(3), 447–456. 10.1177/03635465020300032501 12016090

[CIT0013] Haim A., Yaniv M., Dekel S. & Amir H, 2006, ‘Patellofemoral pain syndrome: Validity of clinical and radiological features’, *Clinical Orthopaedics and Related Research* 451, 223–228. 16788411 10.1097/01.blo.0000229284.45485.6c

[CIT0014] Harvie D., O’Leary T. & Kumar S, 2011, ‘A systematic review of randomized controlled trials on exercise parameters in the treatment of patellofemoral pain: What works’, *Journal of Multidisciplinary Healthcare* 4, 383–392. 22135495 10.2147/JMDH.S24595 PMC3215347

[CIT0015] Heintjes E., Berger M.Y., Bierma-Zeinstra S.M., Bernsen R.M., Verhaar J.A. & Koes B.W, 2003, ‘Exercise therapy for patellofemoral pain syndrome,’ *Cochrane Database* *System Review* 4, CD003472 10.1016/s0031-9406(05)60488-9 14583980

[CIT0016] Heintjes E., Berger M.Y., Bierma-Zeinstra S.M., Bernsen R.M., Verhaar J.A. & Koes B.W, 2004, ‘Pharmacotherapy for patellofemoral pain syndrome.’ *Cochrane Database System Review* 3, CD003470 10.1002/14651858.cd003470.pub2 PMC8276350 15266488

[CIT0017] Lake D.A. & Wofford N.H, 2011, ‘Effect of therapeutic modalities on patients with patellofemoral pain syndrome: A systematic review’, *Sports Health: A Multidisciplinary Approach* 3(2), 182–189. 10.1177/1941738111398583 PMC3445135 23016007

[CIT0018] Lankhorst N.E., Bierma-Zeinstra S.M. & Van Middelkoop M, 2012, ‘Risk factors for patellofemoral pain syndrome: A systematic review,’ *Journal of Orthopaedic & Sports Physical Therapy* 42(2), 81–112. 10.2519/jospt.2012.3803 22031622

[CIT0019] Malanga G.A., Andrus S., Nadler S.F. & McLean J, 2003, ‘Physical examination of the knee: A review of the original test description and scientific validity of common orthopedic tests’, *Archives of Physical Medicine and Rehabilitation* 84(4), 592–603. 12690600 10.1053/apmr.2003.50026

[CIT0020] McCarthy M.M. & Strickland S.M, 2013, ‘Patellofemoral pain: An update on diagnostic and treatment options’, *Current Reviews in Musculoskeletal Medicine* 6(2), 188–194. 23456237 10.1007/s12178-013-9159-x PMC3702777

[CIT0021] Näslund J., Näslund U.B., Odenbring S. & Lundeberg T, 2006, ‘Comparison of symptoms and clinical findings in subgroups of individuals with patellofemoral pain’, *Physiotherapy Theory and Practice* 22(3), 105–118. 16848349 10.1080/09593980600724246

[CIT0022] Nijs J., Van Geel C. & Van de Velde B, 2006, ‘Diagnostic value of five clinical tests in patellofemoral pain syndrome’, *Manual Therapy* 11(1), 69–77. 15950517 10.1016/j.math.2005.04.002

[CIT0023] Nunes G.S., Stapait E.L., Kirsten M.H., De Noronha M. & Santos G.M, 2013, ‘Clinical test for diagnosis of patellofemoral pain syndrome: Systematic review with meta-analysis’, *Physical Therapy in Sport* 14(1), 54–59. 10.1016/j.ptsp.2012.11.003 23232069

[CIT0024] Parker R. & Jelsma J, 2010, ‘The prevalence and functional impact of musculoskeletal conditions amongst clients of a primary health care facility in an under-resourced area of Cape Town’, *BMC Musculoskeletal Disorders* 11(1), 1. 20044944 10.1186/1471-2474-11-2 PMC2830178

[CIT0025] Pećina M. & Bojanić I, 1993, *Overuse injuries of the musculoskeletal system,* CRC Press, Boca Raton, FL.

[CIT0026] Post W.R, 1999, ‘Current concepts clinical evaluation of patients with patellofemoral disorders’, *Arthroscopy: The Journal of Arthroscopic & Related Surgery* 15(8), 841–851. 10.1053/ar.1999.v15.015084 10564862

[CIT0027] Powers C.M., Ho K.Y., Chen Y.J., Souza R.B. & Farrokhi S, 2014, ‘Patellofemoral joint stress during weight-bearing and non-weight-bearing quadriceps exercises’, *Journal of Orthopaedic & Sports Physical Therapy* 44(5), 320–327. 10.2519/jospt.2014.4936 24673446

[CIT0028] Prins M.R. & Van Der Wurff P, 2009, ‘Females with patellofemoral pain syndrome have weak hip muscles: A systematic review’, *Australian Journal of Physiotherapy* 55(1), 9–15. 19226237 10.1016/s0004-9514(09)70055-8

[CIT0029] Rathleff M.S., Roos E.M., Olesen J.L. & Rasmussen S, 2013, ‘High prevalence of daily and multi-site pain – A cross-sectional population-based study among 3000 Danish adolescents,’ *BMC Pediatrics* 13(1),191. 24252440 10.1186/1471-2431-13-191 PMC3840664

[CIT0030] Smith T.O., Davies L., Chester R., Clark A. & Donell S.T, 2010, ‘Clinical outcomes of rehabilitation for patients following lateral patellar dislocation: A systematic review’, *Physiotherapy* 96(4), 269–281. 10.1016/j.physio.2010.02.006 21056161

[CIT0031] Smith T.O., Hunt N.J. & Donell S.T, 2008, ‘The reliability and validity of the Q-angle: A systematic review’, *Knee Surgery, Sports Traumatology, Arthroscopy* 16(12), 1068–1079. 10.1007/s00167-008-0643-6 18841346

[CIT0032] Sweitzer B.A., Cook C., Steadman J.R., Hawkins R.J. & Wyland D.J, 2010, ‘The inter-rater reliability and diagnostic accuracy of patellar mobility tests in patients with anterior knee pain’, *The Physician and Sportsmedicine* 38(3), 90–96. 10.3810/psm.2010.10.1813 20959701

[CIT0033] Waryasz G.R. & McDermott A.Y, 2008, ‘Patellofemoral pain syndrome (PFPS): A systematic review of anatomy and potential risk factors,’ *Dynamic Medicine* 7(1), 9. 18582383 10.1186/1476-5918-7-9 PMC2443365

[CIT0034] Witvrouw E., Lysens R., Bellemans J., Cambier D. & Vanderstraeten G, 2000, ‘Intrinsic risk factors for the development of anterior knee pain in an athletic population a two-year prospective study’, *The American Journal of Sports Medicine* 28(4), 480–489. 10921638 10.1177/03635465000280040701

